# Theory of tailorable optical response of two-dimensional arrays of plasmonic nanoparticles at dielectric interfaces

**DOI:** 10.1038/srep33712

**Published:** 2016-09-22

**Authors:** Debabrata Sikdar, Alexei A. Kornyshev

**Affiliations:** 1 Department of Chemistry, Faculty of Natural Sciences, Imperial College London, Exhibition Road, South Kensington, London, SW7 2AZ, United Kingdom

## Abstract

Two-dimensional arrays of plasmonic nanoparticles at interfaces are promising candidates for novel optical metamaterials. Such systems materialise from ‘top–down’ patterning or ‘bottom–up’ self-assembly of nanoparticles at liquid/liquid or liquid/solid interfaces. Here, we present a comprehensive analysis of an extended effective quasi-static four-layer-stack model for the description of plasmon-resonance-enhanced optical responses of such systems. We investigate in detail the effects of the size of nanoparticles, average interparticle separation, dielectric constants of the media constituting the interface, and the nanoparticle position relative to the interface. Interesting interplays of these different factors are explored first for normally incident light. For off-normal incidence, the strong effects of the polarisation of light are found at large incident angles, which allows to dynamically tune the reflectance spectra. All the predictions of the theory are tested against full-wave simulations, proving this simplistic model to be adequate within the quasi-static limit. The model takes seconds to calculate the system’s optical response and makes it easy to unravel the effect of each system parameter. This helps rapid rationalization of experimental data and understanding of the optical signals from these novel ‘metamaterials’, optimised for light reflection or harvesting.

By virtue of the surface plasmon resonances (SPRs) in metallic or composite nanoparticles (NPs) it has been possible to realise different nanoparticle-based architectures exhibiting numerous exotic optical properties—such as directed narrowband-scattering, resonance-enhanced wide-band absorption, as well as intense near-field confinement and enhancement^1^,^2^,^3^,^4^,^5^,^6^,^7^. These traits enabled plasmonic NPs to feature in a wide variety of applications ranging from light harvesting to sensing, and also from guided transportation of energy to energy localisation for photothermal therapeutics and imaging^3^,^4^,^5^,^6^,^7^,^8^,^9^,^10^,^11^,^12^,^13^,^14^. Each NP enacts a tuneable optical antenna that allows one to tailor the spectral position, width, and intensity of the SPR as functions of the NP’s size, shape, composition, and surrounding medium^15^,^16^,^17^,^18^,^19^. NPs forming a linear chain or a two-dimensional (2D) periodic array can allow further tuning of their collective SPR as functions of their inter-particle spacing and even of lattice orientation when NPs are ordered in a 2D periodic arrangement^20^,^21^,^22^,^23^,^24^. This is typically the scenario for freestanding NP arrays in an isotropic dielectric medium. For NPs positioned in the vicinity of a substrate, the property of the substrate and its distance from the NPs act as additional parameters that could be used to tailor the system optical response^9^,^25^,^26^,^27^,^28^. Such structures offer promising nanoplasmonic applications; in particular as components of variable optical devices^3^,^29^,^30^,^31^ for reflecting or transmitting, as well as harvesting light *at will*. Assembly or stimulated self-assembly, and experimental characterisation of such systems are currently hot subjects of research in several group including ours^32^,^33^,^34^. Therefore, a simple yet adequate theory, capable of describing the role of different system parameters and how the interplay between those could modify the system response, is always in demand to facilitate the rational design of such architectures.

A comprehensive theoretical study of such nanoplasmonic system would provide a platform to design a range of futuristic optical devices where plasmonic NPs immersed in a fluidic (air or liquid) medium could be precisely positioned or self-assembled at or near interfaces of another liquid or solid medium. It means the substrate could possibly be any medium with a different dielectric constant than that of the medium in which the NPs are submerged. In the case liquid/liquid environment NPs could further be piercing the interface, submerging partially in each of the two media. Recently, there has been a huge drive towards designing such systems where plasmonic NPs self-assemble at liquid/liquid interfaces or at the interface of liquid/solid-films (of semiconductors or semi-metals or metals)^35^,^36^,^37^,^38^,^39^,^40^. Involving electrolytes (as, *e.g.* in systems of two immiscible electrolytic solutions, metal/electrolyte, or semiconductor/electrolyte interfaces), voltage can be applied across the interface, acting as an additional and easily/continuously tuneable parameter, which could change the structure of NP arrays and thereby their optical response. This will be opening the way towards switchable mirror/windows, tuneable colour reflectors, and surface enhanced Raman **s**pectroscopy (SERS) based sensors^35^,^36^,^37^.

In this paper we abstract ourselves from how the arrays self-assemble and build the structures of interest, but will present a comprehensive study of optical response of NP arrays in such systems. We will rest the analysis on an extended effective medium theory^36^,^41^,^42^ developed here and improved on several fronts, but also explored in greater detail. The theory itself is based on dipolar approximation of optical response of nanoparticles, takes into account image dipoles emerging at the interface, all incorporated into a multi-layer Fresnel reflection theory.

Previewing, within defined and physical justified limits, our theoretically calculated reflectance spectra, using a four-layer stack model, agree exceptionally well with the ones computed numerically based on full-wave simulations. This testifies correctness and adequacy of this simplistic theory in emulating complex NP systems, and justifies a comprehensive analysis of various factors affecting the reflectivity, based on this model. It takes just seconds on a personal computer to plot one spectrum based on the theory, whereas simulations often take much longer time. Thus, use of the theory allows a ‘feed-back mode’ of the study and effective search for the most important effects.

In the subsequent sections we provide complete derivation of this theory, followed by a detailed analysis of the effect of NP size and lattice spacing, along with the influence of permittivity of a dielectric substrate and its gap from the NP layer. Two different sets of NPs—small ones with radius *R* = 6.25 nm and large ones with *R* = 25 nm, arranged in a hexagonal 2D array with different lattice constants are analysed here for different permittivities of the ‘substrate’ material forming the interface with the NP surrounding medium. With increasing the substrate permittivity the interface itself becomes more reflective, and hence, this study helps to understand the exact role of the substrate material in the overall optical response of the system. Our parametric study provides a wider picture of the dependency of optical reflectance on critical system parameters such as NP size, substrate permittivity, and gap of NP layer from the interface. Furthermore, apart from the normal incidence, the reflectance spectra show strong dependence on the polarisation as well as incident angle of light. This would allow one to dynamically tune the optical reflectance obtained from the same physical system.

We explain physically each highlighted effect and its associated trend, which is one of the goals of this comprehensive paper. This simplistic theory, thus, enables one to rationalize optical properties of a very complicated physical system, often studied through numerical simulations only. The theoretical model and the derived equations could cater to designing of layered NP-assisted solar cells with appropriate choice of materials for different layers in the stacked model. This would allow precise tuning of optical responses besides achieving dynamic control to operate the same system as an optical mirror, window, or switch with variable high or low reflectance to be used in numerous futuristic applications, and even in designing light harvesting devices like solar cells.

## Methods

### Theoretical Model Formulation

**Figure 1:**
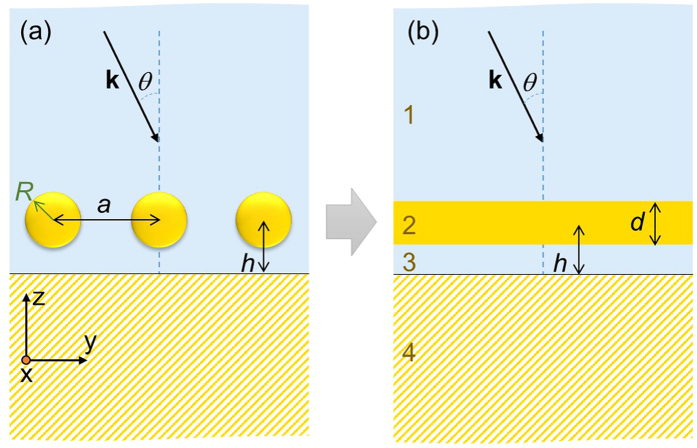
Theoretical model emulating a system of nanoparticles assembled near an interface. Schematic representation of nanoparticles (NPs) arranged as an ordered array immersed in a dielectric medium, which are placed in the vicinity of another dielectric medium considered as substrate. A practical system (**a**) is emulated using a four-layer stack model (**b**) in order to estimate its optical responses. Incident light propagates in layer 1 (having dielectric constant *ε*_1_) with wavevector ***k*** and incident angle *θ*. Layer 2 depicts NP monolayer with characteristic width *d*, which represents an ordered array of metallic NPs, each of radius *R*, with lattice constant *a* and the NP centres positioned at distance *h* from the substrate (layer 4). The NPs are separated from the interface by a spacer shown as layer 3.

In order to calculate the optical response of a layer of NPs assembled near the interface between two dielectric media, as depicted in Fig. 1(a), we adopted a simplified four-layer stack model shown in Fig. 1(b). Previous versions of this model have been reported in refs 35, 36, 37, developed as an extension of the older works^41^,^42^, in particular taking into account contributions from image dipoles. The present article suggests several amendments of the theoretical model, making it more accurate, and systematically compares its results with full-wave simulations. Therefore we choose to describe the model from the scratch in more detail, while commenting on the improvements in due places.

Light impinging on the system is considered to incident from layer 1 (having dielectric constant *ε*_1_) at an angle *θ*, propagating with wavevector ***k***. In Fig. 1(b), layer 2 represents NP monolayer with characteristic width *d*, which emulates the optical properties from an ordered array of metallic NPs, each of radius *R*. The NPs are considered to be arranged in a two-dimensional (2D) array having lattice constant *a* with their centres positioned at distance *h* from the interface *i.e.*, from the surface of the substrate (layer 4). Layer 2 can then be characterised by anisotropic frequency-dependent dielectric tensor with components 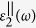 and 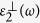, which are derived and discussed in the subsequent paragraphs. Layer 3 (with dielectric constant *ε*_3_) is considered to be the separating layer between the NP layer and the substrate’s surface, and in practice this could represent the NP capping ligands, or a solid dielectric spacer, or even the embedding medium of the NPs. Layer 4 depicts a semi-infinite material (with dielectric constant *ε*_4_) acting as substrate, which represents the other liquid or solid medium participating in forming the interface along with the medium where NPs are immersed.

Note that for a metal, semi-metal or semiconductor medium as the substrate (layer 4), one needs to consider *ε*_4_ to be frequency-dependent; whereas, for a simple dielectric material *ε*_4_ in the UV–visible range could be assumed as a frequency-independent constant. The reflectance spectrum of a four-layer stack system can be calculated by employing Fresnel expressions for multi-layer stacks, but before doing that it is necessary to characterise the effective dielectric function of the metallic NP monolayer, which is represented as layer 2 in Fig. 1(b). At optical wavelengths(*λ*), the dielectric response of a metal is strongly affected by inter-band transitions that demands the Drude (D) permittivity model to be extended using a Drude–Lorentz (DL) model, which takes the following form^37^:


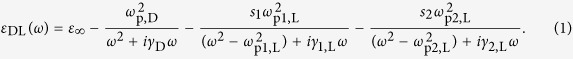


Here 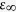 is the permittivity limit at high frequencies, *ω*_p,D_ and *γ*_D_ denote the plasma frequency and damping coefficient from the Drude model, respectively. The third and the fourth term in Equation (1) are the two additional Lorentzians (L) with resonance frequencies *ω*_p1,L_ and *ω*_p2,L_, where *γ*_1,L_ and *γ*_2,L_ represent the spectral widths of the two resonances with *s*_1_ and *s*_2_ being their weighting factors. Although the basic formalism can be applied for any material of NPs, in this paper we focus only on gold NPs and to model the permittivity of gold, we chose the parameters listed in Table 1 which make the approximation in Equation (1) to closely fit the experimental data^43^:

The next step is to express the quasi-static polarisability *α*(*ω*) of a sub-wavelength spherical NP with radius *R*(≪*λ*) through bulk permittivity of its material and the dielectric constant of the medium in which the NP is embedded. Using a classical dipolar approximation


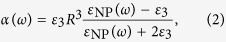


where the NP, with permittivity 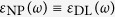, is considered to be embedded in a medium of permittivity *ε*_3_. When such NPs are arranged in hexagonal or square lattice forming a quasi-2D layer, the effective dielectric permittivity *ε*_2_(ω) of such layer can be given by


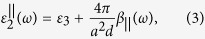



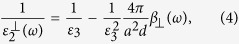


where 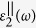 and 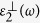 are the parallel and perpendicular components of the dielectric tensor, *a* is the lattice constant of the 2D array, and *d* is the characteristic thickness of the emulated NP monolayer. Here 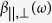 represent the effective polarisability of each NP interacting with all other NPs in the layer and also with their images in the substrate medium, estimated within the scope of dipolar approximations.

For a layer of NPs forming a 2D array, being embedded in a dielectric medium with permittivity *ε*_3_ and positioned in the vicinity of a substrate having frequency-dependent permittivity *ε*_4_(*ω*), the effective quasi-static polarisability of each NP in the array can be expressed as


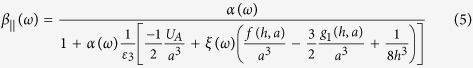



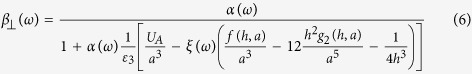


Here, *α*(*ω*) is the isotropic polarisability of each individual free-standing NP given in Equation (2) and 
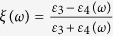
 is the image-charge screening factor. The lattice dependent parameter *U*_*A*_ and the functions —*f*(*h*, *a*), *g*_1_(*h*, *a*) and *g*_2_(*h*, *a*)— are calculated from the lattice sums:

For a square lattice —


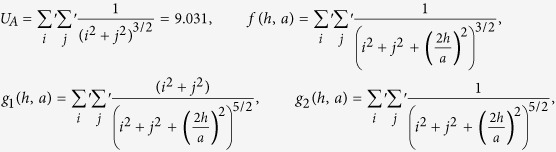


For a hexagonal lattice —


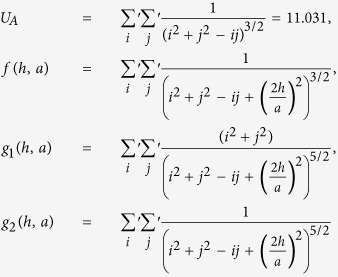


In Equation (5) the second term in the image contributions for 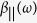 is corrected w.r.t. to similar term in Equation (12) of ref. 36. In equation (6) the second and third terms in the image contributions for 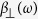 have also been revised, replacing the similar terms in Equation (13) of ref. 36. Note that in 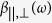 expressions, *U*_*A*_ sums up the contributions from all NPs in the monolayer interacting with any given NP, *f*(*h*, *a*), *g*_1_(*h*, *a*) and *g*_2_(*h*, *a*) contribute towards adding up the effects arising from images of the all ‘other’ NPs, whereas the last term with 1/*h*^3^ dependence incorporates the effects from the NP’s own image. The intensity of the obtained optical response can be related to 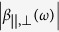.

It is important to note that though a freestanding individual NP’s polarisability in the bulk is isotropic, the effective polarisability of every individual NP forming a 2D array and/or just placed in the vicinity of a flat interface is no longer isotropic. The NP experiences substrate-induced anisotropy in its dielectric response. Consequently, the effective dielectric function of the NP layer is anisotropic. With no off-diagonal components, it has three components—one perpendicular (⊥) and two parallel (∥) to the interface. As seen from the above equations (Equations (5) and (6)), the magnitude of 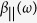 and 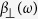 are strongly dependent on the composition and size of the NPs, and to their lattice spacing. It also depends on the order of the 2D array arrangement (structure of the layers, local disorder, *etc.*), as well as on the separation between the NPs and the interface.

With the knowledge of dielectric permittivity of each layer, the transfer matrix 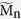 that relates the reflection (*r*) and transmission (*t*) coefficients from each interface *n* between layers *n* and *n* + 1 is given by,


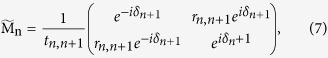


with 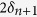 being the phase difference between the two reflected waves in layer *n*: one reflected straightaway from the interface between layer *n* and *n* + 1; and another gets transmitted through that interface, passes through layer *n* + 1, gets reflected from the next interface and then returns by passing through layer *n* + 1 again to be finally transmitted in layer *n*.

Therefore, for a four-layer stack system, which has three interfaces, the transfer matrix is calculated as 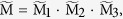 which can be expressed in the form:


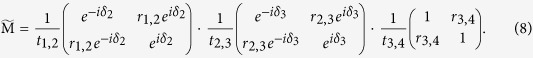


The overall reflection coefficient from such system is then given by 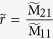, where 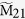 and 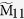 are the elements of the final transfer matrix given in Equation (8), and therefore 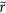 can be deduced to


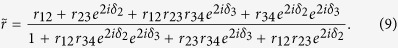


However as discussed above, in the case of metallic NPs the NP layer (layer 2) is characterised by anisotropic dielectric permittivity tensor with different components in parallel and perpendicular direction of the incident electric field. This implies the Fresnel reflection coefficient would be different for s- and p-polarised excitation of incident light. Therefore, the reflection coefficient can better be explicitly written as


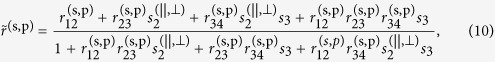


where 
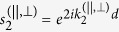
 and 
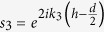
 are the phase factors, 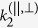 are the parallel/perpendicular components of the wave vector in layer 2, *k*_3_ denotes the wave vector in layer 3, and *r*_*ij*_ is the reflection coefficient at the *i*/*j* interface given by


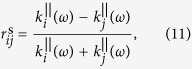


and


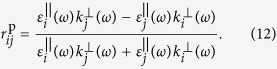


The wave vectors in Equations (11) and (12) can be obtained as:


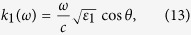



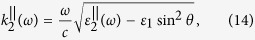



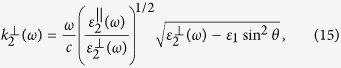



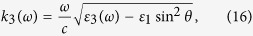



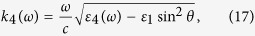


where 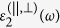 are the parallel and perpendicular components of the dielectric constant of layer 2, and 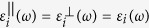 for *i* = 1, 3, and 4. This theoretical approach thus allows us to calculate the reflectance *R*^(s,p)^ from the four-layer stack system in form of 
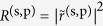
. In a similar manner the transmittance *T*^(s,p)^ through this system can be expressed as 
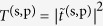
, where 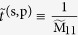 is explicitly of the form:


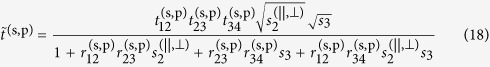


with 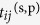 being the transmission coefficient at the *i*/*j* interface for s- and p-polarised light given by


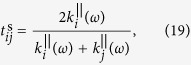


and


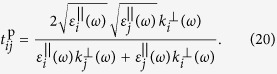


Using the expressions for the reflectance and the transmittance one can calculate the absorbance (*A*) of light in such four-layer system using the relation: 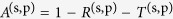. The results obtained based on this theory are discussed in the subsequent sections.

## Results

Based on the above described theory, we now investigate spectra of optical response from a system of gold NPs immersed in water, where the NPs are assembled forming a hexagonal array in the vicinity of a dielectric substrate. We calculate the spectra using the quasi-static dipolar approximation and the effective medium theory. The latter expresses the optical polarisability of a NP-array through effective dipolar response of individual NPs influenced by ‘bonding’ or ‘anti-bonding’ type interactions^44^ with the dipolar modes of all other NPs, with its own dipolar image, and with the dipolar images of the all other NPs. Though the effects coming from all other NPs arranged in the two-dimensional lattice are summed up, the main contributors would always be the neighbouring NPs as the coupling strength between NPs diminishes with increasing distance.

**Figure 2:**
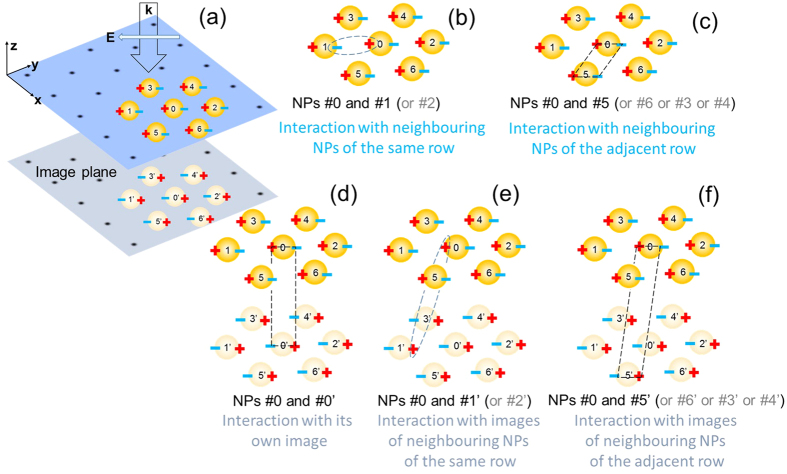
Schematic representation of different scenarios of plasmonic interaction in a hexagonal array of nanoparticles in the vicinity of a substrate. (**a**) Schematic of a set of nanoparticles (NPs) positioned on hexagonal lattice points. As the strongest interactions come from the nearest neighbours, a set of seven NPs are thus chosen here for demonstration of the concept. Dipolar surface charges get induced on the NPs due to polarisation by the incident electric field (E). This also induces image charges of these NPs on the ‘image plane’, here in the substrate. ((**b**–**f**)) Different plasmonic interaction scenarios for any individual NP (say, NP #0): interaction with (**b**) NPs in the same row, (**c**) NPs in the adjacent rows, (**d**) its own image, (**e**) images of neighbouring NPs in the same row, and (**f**) images of neighbouring NPs in the adjacent rows. Like-charges give rise to ‘anti-bonding’ type coupling whereas opposite charges experience ‘bonding’ type coupling. Note that, this schematic and depicted types of coupling hold for substrates with permittivity larger than the NP embedding medium. Only then each charge will have an image of opposite sign.

In such complex multi-NP–multi-image interaction scenario the dominating type of interaction decides the trend of the spectral shift of plasmonic resonance in the optical response. This shift can be interpreted by analysing bonding and anti-bonding type plasmonic interactions based on the well-established plasmon hybridization theory^44^. The type of interaction between an NP and its neighbours in the hexagonal array as well as with its own image and the images of its neighbouring NPs (see Fig. 2(a)) depends strictly on the parameters such as, the array’s lattice-constant, spacer thickness, and radius of an individual NP. Due to the polarisation induced by the incident electric field, each NP assumed to behave like an induced dipole, always experiences bonding-type interaction with its neighbouring NPs positioned in the same row (see Fig. 2(b)). This is because the induced charges on the surface of a NP could find their opposite charges much closer (as compared to their same-sign charges) on the surface of the neighbouring NPs from the same row, leading to an overall bonding-type interaction. With lattice spacing getting larger, the bonding type interaction weakens; however for fixed lattice spacing this interaction strengthens with increasing size of NPs.

Additionally, there are interactions from other neighbouring NPs, positioned mainly in the adjacent rows (left and right rows) of the monolayer. The charges on each neighbouring NP in the adjacent row may be assumed to be placed at the corners of a parallelogram joining the NP’s surface charges (see Fig. 2(c)). With little calculations it is found that the overall interaction from each of these neighbouring NPs of the adjacent rows is of anti-bonding type, and the associated potential energy scales as *R*^2^/*a*^3^. Therefore, for a fixed lattice constant, anti-bonding type interaction gets stronger for larger NPs; whereas, at a fixed NP size, the anti-bonding interaction gets weaker with increasing lattice constant.

Not to forget, there is a significant contribution from image charges as well, especially at very thin spacer thickness. In this case, the NP feels strong bonding interaction with its own image (see Fig. 2(d)). For a fixed spacer thickness, the relative contribution of image effect gets stronger for smaller NPs. With images of the neighbouring NPs in the same row (see Fig. 2(e)), the NP experiences anti-bonding interaction; whereas images of NPs from the adjacent rows contribute to a bonding-type interaction (see Fig. 2(f)). The strength of interaction from the images of neighbouring NPs depend on both spacer thickness and lattice constant. However, such interaction is usually weaker than the effects coming from NP’s own image or from its adjacent NPs due to the larger distance involved.

Throughout this paper we assumed *ε*_1_ = *ε*_3_ = 1.78 (which corresponds to water), set the characteristic dimension of the dipole layer to *d* = 1 nm 36, and considered layer 4 to be composed of a dielectric material with frequency-independent dielectric function *ε*_4_. Where pertinent, we compare the results with full-wave numerical solutions of Maxwell’s equations for the same system. Two different sets of NPs — small and large having 6.25 nm and 25 nm radius, respectively, — are considered for the investigation.

**Figure 3:**
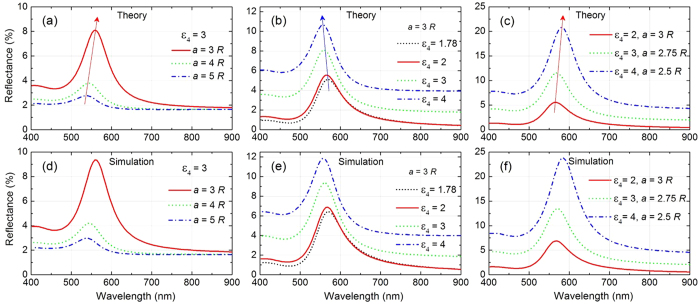
Effects of lattice spacing and substrate permittivity on optical reflection. Reflectance spectrum calculated based on theory (**a–c**) and obtained from numerical simulations (**d–f**) as function of lattice constant (**a,d**) for a fixed substrate (layer 4) permittivity *ε*_4_ = 3, as function of layer 4 permittivity *ε*_4_ (**b,e**) for a fixed lattice constant *a* = 3*R*, and as function of both *ε*_4_ and lattice constant *a* (**c**,**f**). In all cases, radius of all gold NPs arranged in a 2D hexagonal lattice is considered to be *R* = 6.25 nm, the gap between NP layer and layer 4 substrate is fixed at 1 nm and *ε*_1_ = *ε*_3_ = 1.78.

In both cases, the theory matches simulations exceptionally well (see Fig. 3 (for small NPs) and Supplementary Fig. S2 (for large NPs)). The match is even better for the large NPs. For a fixed ratio of lattice constant to radius, larger size of NPs ensures wider separation between them, making dipolar approximations more accurate. Only when the NPs are very densely packed in the arrays with inter-NP spacing of about 1–2 nm, hybridized multipolar modes become important, and this is when this theoretical model loses its accuracy to some extent. This is seen from Supplementary Figs S1 (small NPs) and S3 (large NPs). With inter-NP spacing of 3–4 nm or beyond, the theoretical curves practically coincide with the simulation ones. This is most encouraging as this portraits the typical situation, when each NP is capped with ligands of typical lengths of 1.5–2 nm, thus restricting inter-NP spacing to go below 3–4 nm.

On the other hand the coincidence between the theory and simulation worsens a bit for very low density arrays (see Supplementary Fig. S4). In this limit our continuum film approximation emulating the array is not very accurate, strictly speaking. However, as long as the distance between NPs is still smaller than the wave-length of the incident light, the qualitative correspondence between the theory and simulation remains good. Note that for very dilute arrays the system loses its practical importance so that the loss of accuracy of the theory in this limit is irrelevant. Even for the arrays of very large NPs (radius of 40 nm), the plots (shown in Supplementary Fig. S5) indicate reasonable accuracy and effectiveness of our simplistic theoretical model, both for very high and low array densities.

## Optical reflectance calculated as function of system parameters

Figure 3(a) depicts the reflectance spectrum from an array of the small gold NPs (*R* = 6.25 nm) as a function of lattice constant *a* when the array is positioned 1 nm away from a dielectric substrate with permittivity *ε*_4_ = 3. With reducing lattice constant the NPs get closer to each other and hence, the interaction between their localised plasmon modes gets stronger resulting in dramatic increase in peak reflection along with the red-shift in the peak wavelength *λ*_max_. The theoretical results match very well with the ones calculated numerically using COMSOL Multiphysics^®^, shown in Fig. 3(d). The reflectance values obtained from the numerical simulations are slightly higher than those calculated theoretically. This minor difference is not unexpected. Indeed this simplistic theory rests on dipolar approximation used for the description of polarisability of NPs, whereas in reality there are some contributions from higher order multipoles^45^,^46^ which are taken into account in the numerical simulations. To the accuracy of this minor difference, this theory reproduces the reflectance of such complex system very well, which allows one to readily investigate such system’s optical responses as function of different system parameters.

Figure 3(b,e) present the reflectance spectral profiles obtained from theory and simulations, respectively, at different values of substrate (layer 4) permittivity *ε*_4_ (for a fixed lattice constant *a* = 3*R*). For liquid/liquid interfaces *ε*_4_ typically lies below 3 for commonly known solvents. However, for solid substrates as typically used in light harvesting applications *ε*_4_ could be even larger than 4 (*e.g.*, ZnSe has permittivity of 7.5). The range of *ε*_4_ values used in this study is sufficient to show the relevant trends.

It is seen in both cases that with increase in *ε*_4_ there is increase in reflectance, but with a trend of blue-shift for *λ*_max_. This counterintuitive effect has a rather subtle nature. Remember that in a 2D array of NPs at the interface, each NP interacts with its neighbouring NPs as well as the images of its own and of its neighbours, and the type of dominating interaction decides the trend. When anti-bonding type of interactions (of a NP with the neighbouring NPs from adjacent rows and from the images of the neighbouring NPs of the same row) overpower the bonding type interactions (with neighbouring NPs, with its own image and also images of the NPs of the adjacent rows), the reflectance peak exhibits blue-shift. Both the theory and simulations (see Fig. 3(c,f)) show that this blue-shift could be compensated by bringing the NPs closer. Indeed, with increasing *ε*_4_ and simultaneous reduction in lattice spacing *a*, the reflectance peak exhibits red-shift. The increase in reflectance in each case with increasing *ε*_4_ could be attributed to the interplay of the increasing background reflection from the bare interface at larger *ε*_4_.

**Figure 4:**
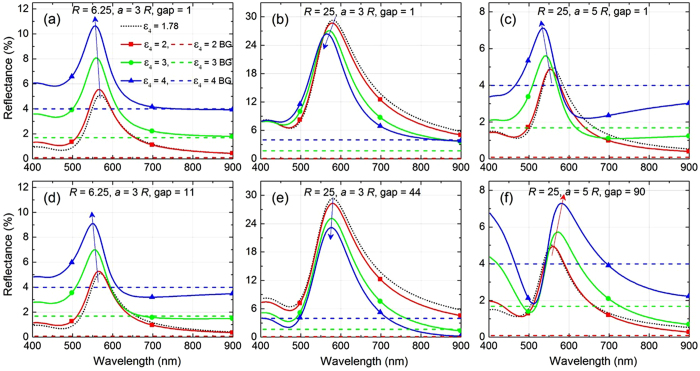
Effects of substrate permittivity on optical reflectance at different lattice spacing and different gaps of nanoparticle layer from substrate. Reflectance spectra calculated based on theory depicting the effects from a dielectric substrate (layer 4) in terms of its permittivity (*ε*_4_) and distance (or gap) from NP monolayer (layer 2). Parameters: (**a,d**) *R* = 6.25 nm and *a* = 3*R* having gap of 1 nm (**a**) and 11 nm (**d**), (**b,e**) *R* = 25 nm and *a* = 3*R* having gap of 1 nm (**b**) and 44 nm (**e**), and (**c,f**) *R* = 25 nm and *a* = 5*R* having gap of 1 nm (**c**) and 90 nm (**f**). In every Figure, the dashed lines represent the background (abbreviated as BG) reflection with no NP layer present, calculated for each value of *ε*_4_. Here, we consider normal incidence of light *i.e.*, 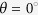 and *ε*_1_ = *ε*_3_ = 1.78.

Figure 4(a) depicts the reflectance spectra for different *ε*_4_ at a fixed lattice spacing, similar to the one shown in Fig. 3(b) but along with background (abbreviated as BG) reflectance arising from the plain interface without NPs (shown using dashed horizontal lines) for each *ε*_4_. This allows one to appreciate the fact that due to increase in the background reflectance with *ε*_4_, the overall reflectance increases. However, with increasing *ε*_4_ the image-charge factor also gets stronger in magnitude. This factor, being of opposite sign, tends to reduce the effective dipole moment of the NPs, which results in reduction in reflectance. In case of small NPs, like those of *R* = 6.25 nm radius in Fig. 4(a), this latter effect is not strong enough to overpower the basic increase of reflection from the dielectric–dielectric interface. The former effect overpowers the latter and results in an overall increase in reflectance, as can be witnessed in case of small NPs in Fig. 4(a). On the contrary, for larger NPs of *R* = 25 nm (see Fig. 4(b,e)) the latter effect dominates. The image contributions reduce the effective induced dipole moments of NPs, which leads to an overall reduction in reflectance with increasing *ε*_4_ upon compensating the increase in reflectance coming from the bare interface that gets more reflective at larger *ε*_4_.

The lattice size effect is straightforward. In Fig. 4(c), where the lattice spacing has increased to *a* = 5*R* from that of *a* = 3*R* in Fig. 4(b), for the same large size of NPs, we see that the reflectance dramatically decreases. With large lattice spacing, reflectance from NP layer becomes comparable to that from the background (like we would have had, if we were diminishing the size of NPs). Not surprisingly, here again we witness an increase in overall reflectance with *ε*_4_, as is observed in Fig. 4(a), because the contribution of NPs themselves is so small here. It is noteworthy that despite the changes in overall reflectance, there is a trend of blue-shift in 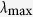 seen in all cases (see Fig. 4(a–c)). This is well understood from the explanation given above for the blue-shift observed in Fig. 3(b). Note that in all these cases, the interaction of dipolar excitations in NPs with the image dipoles induced in the substrate is very strong as the gap between NP layer and the substrate layer 4 is considered to be as small as 1 nm. It would be interesting to further explore if these trends remain the same when the effects from the substrate gets weaker at larger gaps.

The lower panel in Fig. 4 depicts the scenarios at larger gap between NP layer and the substrate. At larger gap, the substrate-induced effects would be comparatively weaker and might exhibit different trends, and hence, deserves further investigation. Figure 4(d) presents the case similar to Fig. 4(a), but for a larger gap of 11 nm. It can be seen that in such cases of comparatively weaker substrate interaction the overall reflectance is much lower as compared to Fig. 4(a) where gap is just 1 nm. Reflectance at larger wavelengths gets even lower than the background reflectance, which may be either attributed to increased absorbance of light, reflected from the interface, by the NPs located at a substantial gap from the interface or to the phenomenon of enhanced transmittance (discussed in the next sub-section). Similar observation of reduction in overall reflectance is made for large NPs with 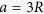 and gap =44 nm in Fig. 4(e).

Moreover in Fig. 4(e), at significantly large gap of 44 nm the anti-bonding type interaction of a NP with the images of neighbouring NPs of the same row, which is responsible for the blue-shift of 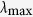, gets much weaker. Therefore, the blue-shift trend looks diminishing at larger gaps with the substrate. This can be further appreciated in Fig. 4(f), where the same NPs are arranged with larger lattice spacing *a* = 5*R*, the gap has been considered to be as large as 90 nm. At such large gap and larger lattice spacing, it is expected that the bonding type interactions between NPs and with its own image would be much stronger than the anti-bonding type interactions rising from images of the neighbouring NPs of the same row. Thus one could see the trend of red-shift in 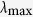 in this case. The overall increase in reflectance could be well understood using the same arguments that explained Fig. 4(c). However, there are certain special features in Fig. 4 which need further investigation. For instance, the reflectance at long wavelengths from the system with NP layer with larger lattice spacing and/or large gap from the substrate drops below that from the bare interface. To find out the physical reason behind such quenching of reflection, a detailed analysis involving also transmission and absorption is therefore presented below.

## Comprehensive study of different components constituting the optical responses

**Figure 5:**
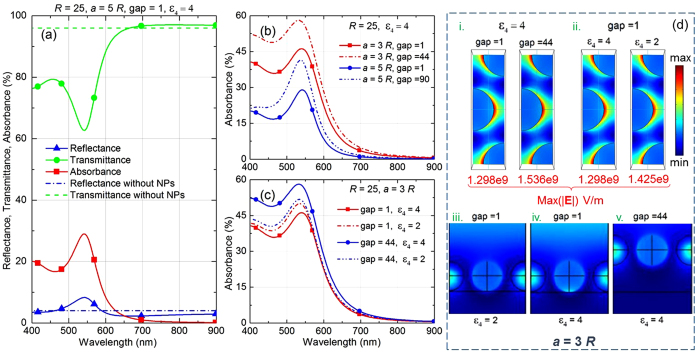
Effects of gap and substrate permittivity on different components constituting optical response of the nanoparticle system. (**a**) Reflectance, transmittance, and absorbance spectra calculated numerically to analyse the effects from a dielectric substrate (layer 4) with and without the presence of NPs. Parameters: *R* = 25 nm, *a* = 3*R*, and gap of 1 nm. (**b,c**) Absorbance spectra for the NP layer (**b**) with different lattice spacing and gap, while substrate permittivity and NP radius are kept constant, and (**c**) with different gap and substrate permittivity, while lattice constant and NP radius are kept constant. In all cases, we consider normal incidence of light *i.e.*, 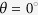 and 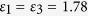. (**d**) Near-field distribution patterns calculated along the horizontal centre plane of the NPs in a hexagonal array (top-view in i and ii), as well as along the vertical plane passing through the centre of the NP in the middle (side-view in iii–v).

Considering a specific case of Fig. 4(c), with *R *= 25 nm, *a* = 5*R*, gap of 1 nm and *ε*_4_ = 4, Fig. 5(a) presents the reflectance, transmittance, and absorbance spectra calculated numerically. It is found that at wavelength above 650 nm, the transmittance of the system with NPs exceeds the transmittance from the bare interface without NPs. In this spectral range the absorption from the NPs do not play a very significant role as absorbance diminishes at such long wavelengths. We refer to this excess transmission phenomenon as ‘enhanced transmission’.

Note that the enhanced transmission observed here is not exactly analogous to the well-known extra-ordinary transmission (EOT)^47^,^48^,^49^ effect, where transmission gets extraordinarily large at some specific resonant wavelength. EOT is generally attributed to the presence of plasmon resonances and constructive interference. In other words, EOT originates from coupling of light with the evanescent waves or plasmon excitations, typically in a metal film with periodically patterned holes in it^47^. In our case, NPs arranged in a 2D periodic array emulate an inverse structure of a perforated metallic film. Within the considered spectral window we do not see an EOT resonance at a specific wavelength, but rather observe moderately enhanced transmission occurring over a wider spectral range. Thus, here we seemingly deal with the effect of far-field constructive interference from the NP array (with minimal contributions from the resonance of the evanescent surface waves), which can be held responsible for this marginally extra transmission over a certain spectral window.

However, it is interesting to notice that absorbance also peaks around the same wavelength where reflectance peak is located. Therefore, absorbance could play a significant role in deciding the peak reflectance. We further study the absorbance spectra as function of lattice constant and gap between central plane of the NP layer and the top surface of the substrate. Figure 5(b) shows that for a fixed substrate permittivity *ε*_4_ = 4, the absorbance peak gets stronger for larger gap in both cases of 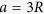 and 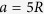, which signifies that a NP-layer absorbs more at large distance from the substrate. This can be attributed to additional absorption of light reflected from the interface, besides absorption of the light directly incident on the NP layer.

Indeed, Fig. 5(c) depicts a particular case of fixed lattice spacing 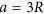 and gap of 44 nm, where absorbance spectra is plotted for different substrate permittivity *ε*_4_. It can be seen that at substantially large gap of 44 nm, with increase in *ε*_4_ the absorbance increases. This testifies the claim that additional absorption comes from light reflected from the interface. At larger *ε*_4_, light is reflected more from the interface, thus allowing the NP layer to absorb more. This is in contrast to the case of a small gap, as low as 1 nm. Here, with the increase of *ε*_4_, absorption reduces. This can be attributed to the fact that electric field gets more localised or confined^4^ in the gap between NPs and the substrate layer for a larger permittivity substrate.

Study of the near-field distribution patterns could help one to appreciate the above facts. Though our theory does not provide a direct estimate of the electric field confinement factor, here we calculated this factor through numerical simulations, similarly to how it is done by other groups^50^. The near-field distribution in Fig. 5(d.i) shows that for fixed lattice constant and substrate permittivity the maximum electric field magnitude, Max(|**E**|), around the NPs gets stronger at larger gap, which indicates stronger absorption by the NPs. Figure 5(d.ii) shows that for small gaps the electric field strength around NPs reduces in the case of a substrate of larger permittivity. This reduction is seemingly due to a stronger concentration of the field confined in the gap (compare Figs 5(d.iii) and (d.iv)). Near-field pattern at large gap of 44 nm in Fig. 5(d.v) is also provided for reference.

**Figure 6:**
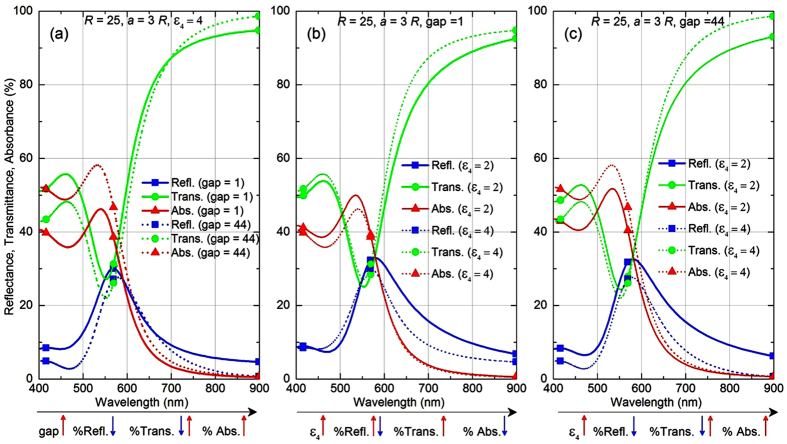
Detailed comparison among different components of optical responses with varied system configurations. (**a**) Reflectance, transmittance, and absorbance spectra calculated numerically to analyse the effects due to (**a**) gap *i.e.*, the distance of NP layer central plane from dielectric substrate (layer 4) with permittivity 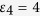, (**b,c**) the substrate permittivity 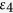 for fixed gap of (**b**) 1 nm and (**c**) 44 nm. In all cases, we consider normal incidence of light *i.e.,* 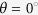 and 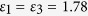. NPs have radii of 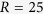 nm and lattice constant 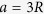.

With the discussion so far, it is evident that to describe the effect of substrate permittivity and of the gap (between the NP layer and the substrate), it is necessary to evaluate the fractions of incident light responsible for all three phenomena—reflection, transmission, and absorption. Figure 6 shows the influence of the gap and of the substrate permittivity *ε*_4_ for fixed gaps on these optical properties for a system of NPs having radii of *R* = 25 nm and lattice constant *a* = 3*R*. The variations at different gaps are shown in Fig. 6(a) for fixed substrate permittivity of *ε*_4_ = 4. It is seen that with increase in gap, the reflectance drops over almost the entire spectral range expect for 600–650 nm window. Transmittance also drops over spectral window of 400–700 nm, beyond which the trend reverses. All these effects happen as there is significant increase in absorption at larger gap over entire wavelength range, specifically for wavelengths around 540 nm where the plasmonic resonance of the NPs is spectrally located. In other words, at shorter gap between the substrate and the NP layer central plane (within the decay length of the evanescent waves of NPs) helps in increasing both reflectance and transmittance within the visible spectrum (400 nm to 700 nm). At larger gap, added absorbance (as NPs now additionally absorb the light reflected from the substrate’s surface) is responsible for simultaneous reduction in reflectance and transmittance. Transmittance at wavelength above 700 nm overcomes that loss due to enhancement in transmission originating from the far-field constructive interference effect.

The effect of *ε*_4_ is further investigated in detail for the above two scenarios of short and large gaps between the NP layer central plane and substrate. When the NP layer is located very close (gap of 1 nm) to the substrate, there would be effects arising from evanescent fields of the NPs penetrating the substrate. This effect is expected to grow with increasing permittivity of substrate. Therefore with increase in *ε*_4_, the reflectance increases slightly for shorter wavelengths till ~550 nm. In this spectral range even transmittance tend to increase, as electric field localisation in the substrate and in the gap increases with *ε*_4_. However, beyond this wavelength, the reflectance gets sufficiently lesser than in the case for lower *ε*_4_. This can be attributed to the significant increase in transmittance with *ε*_4_ at long wavelengths. Notice that, the absorbance experiences an opposite trend with slight drop at long wavelengths, but significant drop at shorter wavelengths near the plasmon resonance wavelength of the NPs. So, it can be said that in the presence of a higher permittivity substrate more light escapes into the substrate over entire spectral range.

However, at larger gap these trends get completely reversed with increase in *ε*_4_, as shown in Fig. 6(c). Evanescent waves of NPs hardly can sense the substrate at such a large gap of 44 nm, and this results in drop of transmittance at short wavelengths with increasing *ε*_4_. The improvement seen in transmittance with *ε*_4_ however persists at long wavelengths as it is solely attributed to the ‘enhanced transmission’ at larger *ε*_4_, which is not dependent on the gap. Reflection drops significantly at large *ε*_4_ over the entire spectral range. The drop in reflectance can be associated with the significant increase in absorbance over the entire spectral range. This is definitely not due to any effects from evanescent wave of NPs near substrate at such large gap, but due to the fact that the NPs now can absorb more light through reflections coming from the surface of the substrate.

## Parametric analysis of optical reflectance maximum and its corresponding wavelength

**Figure 7:**
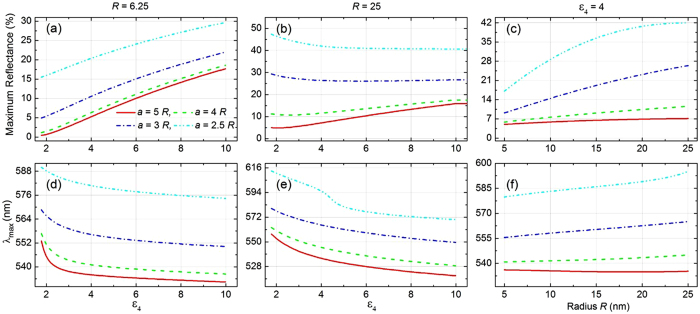
Trends of reflectance maximum and its corresponding wavelength as functions of substrate permittivity and nanoparticle size. Theoretically calculated maximum reflectance (**a–c**) and the wavelength of the maximum reflectance 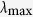
(**d–f**) as a function of layer 4 permittivity *ε*_4_ (**a,b,d,e**) and different lattice constant for NP radius of 6.25 nm (**a,d**) and 25 nm (**b,e**), and as function of NP radius *R* (**c,f**) for fixed *ε*_4_ = 4. In all cases, we consider normal incidence of light *i.e.,* 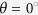, gap = 1 nm and 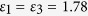.

A bigger picture of substrate’s influence on the reflectance maximum and its peak wavelength 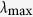 is presented in Fig. 7(a,d) as functions of the substrate permittivity *ε*_4_ at different lattice spacing of NPs in the 2D hexagonal array. The features that we discussed above are all manifested here.

Figure 7(a) shows that for small NPs with radius *R* = 6.25 nm the maximum reflectance monotonically increases with *ε*_4_ irrespective of lattice spacing. The shorter the lattice spacing, the stronger the reflectance. Simultaneously, 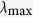 is found to exhibit continuous blue-shift with *ε*_4_, as shown in Fig. 7(d). However, this effect is more pronounced for larger lattice spacing with significantly steeper slope at smaller values of *ε*_4_. The trends are somewhat different and get more interesting for large NPs with radius *R* = 25 nm. As seen in Fig. 7(b), the maximum reflectance increases with *ε*_4_ only at large lattice spacing of *a* = 4*R* and beyond. But for smaller lattice spacing such as *a* = 3*R* or below, the maximum reflectance reduces with *ε*_4_ where reduction in effective dipole moment of NPs, arising from increasing strength of counter-acting image dipoles, becomes dominant. At large lattice spacing such as *a* = 4*R* and beyond, the rise in background reflectance level from the interface helps to overcome the decrease in reflectance due to image contributions and hence, an overall increasing trend of reflectance can be seen with increasing *ε*_4_. The peak wavelength 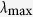, however, appears to exhibit a gradual blue-shift with *ε*_4_, with a steeper slope for larger lattice spacing (see Fig. 7(e)).

We further investigated the variation in maximum reflectance and peak wavelength as functions of NP radius for fixed parameters of lattice spacing and substrate permittivity *ε*_4_ = 4 (see Fig. 7(c,f)). An overall trend of increasing reflectance maximum is observed with growing NP size at different lattice spacing (see Fig. 7(c)). Particularly at shorter lattice spacing of *a* = 2.5*R* the effect is most prominent where highly intense inter-particle coupling prevails, which is dependent on both inter-particle separation and NP size. In this study, as the ratio of lattice constant *a* to the NP radius *R* is kept constant, the inter-particle separation increases linearly with *R*. Therefore there is exciting interplay between the effects of simultaneous increase in both size of the NPs and their inter-particle spacing. At short lattice spacing of *a* = 2.5*R*, for moderately large NP radius (say till 20 nm) strong inter-particle coupling results in dramatic enhancement in reflectance maximum. But beyond this size, the effects of increase in inter-particle separation starts counteracting the effects of increased NP size and hence, the maximum reflectance tends to saturate. At larger lattice spacing, this interplay is not very prominent. An overall trend of increasing reflectance maximum can be witnessed with NP size, but with reduced slopes as the lattice constant gets larger.

Besides the trends of maximum reflectance, the shift in the corresponding wavelength, *λ*_max_, is depicted in Fig. 7(f) as a function of the NP size. At lattice spacing of *a* = 4*R* and below, one can see a gradual trend of red-shift in *λ*_max_ with increasing *R*. The slope of the curves gets steeper at shorter lattice spacing. This prominent red-shifting trend is attributed to strong bonding-type coupling between an NP and its neighbours in the monolayer as well as with its own image. However, at very large lattice spacing, like in the case of *a* = 5*R*, there is a trend of slight blue-shift with increasing radius of the NPs (see Fig. 7(f)). For *a* = 5*R* the separation between two neighbouring NPs is 3*R*; whereas with spacer thickness of just 1 nm the separation between a NP’s surface charges and their images is merely 2*R* + 1 nm. This makes the interaction of a NP with its own image to be the dominant source of bonding interaction in this case. However, with increase in NP radius this bonding gets weaker. As discussed previously, an overall trend of anti-bonding interaction (arising from interactions with the neighbouring NPs of the adjacent rows and from the images of neighbouring NPs of the same row) comes into picture with increasing NP radius at fixed lattice constant, when bonding interaction between NPs in the same row is not that strong. This explains the trend of minor blue-shift in Fig. 7(f) with increasing NP radius for *a* = 5*R*.

## The effects of the angle of incidence and polarisation

**Figure 8:**
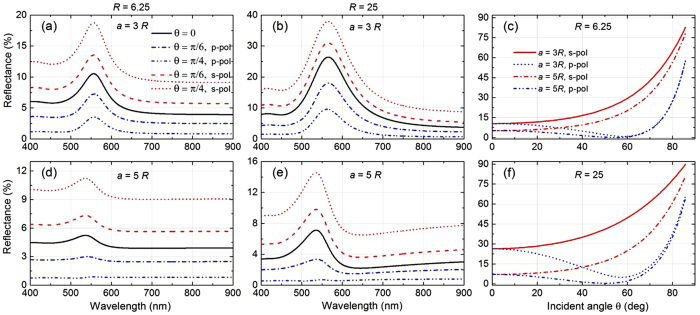
Influence of incident angle and polarisation of light on optical reflectance spectrum and reflectance maximum. Theoretically calculated reflectance spectra (**a,b,d,e**) at different incident angle *θ* for NP radius *R* = 6.25 nm (**a,d**) and *R* = 25 nm (**b,e**) having lattice constant *a* = 3*R* (**a,b**) and *a* = 5*R* (**d,e**). Maximum reflectance calculated as function of light incident angle *θ* for NP radius *R* = 6.25 nm (**c**) and *R* = 25 nm (**f**) having different lattice spacing and polarisation. In all cases, we consider *ε*_1_ = *ε*_3_ = 1.78, *ε*_4_ = 4, and the gap between NP layer and substrate layer 4 as 1 nm.

So far we have considered only normal incidence of light, which makes reflectance from the NP layer independent of light polarisation as both s- and p-polarised light at normal incidence undergo identical reflection. Here, we explore the variation in reflectance from the same system as function of incident angle *θ.* For any non-zero incident angle, light with s-polarisation (also called TE—transverse electric) and p-polarisation (also called TM—transverse magnetic) be reflected differently, *c.f.* Equation (10). Figure 8(a) shows the reflectance spectra at different incident angles for NP layer with *a* = 3*R* and NP radius *R* = 6.25 nm. In all cases the central plane of the NP layer is positioned 1 nm away from the dielectric substrate with permittivity *ε*_4_ = 4. It is seen that with increasing *θ* the reflectance of light increases for s-polarised light, whereas for p-polarised reflectance it reduces, though 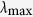 remains unaltered. This feature implies that for a fixed system of NPs, one can simply tune the amount of reflectance just by tailoring the incident angle. Thus switching between high/low reflecting states can be achieved from the same NP array by simply altering the polarisation of the incident light which is identical to the classical laws of Fresnel reflection from a bare interface without NPs. This feature makes such NP-based optical system dynamically tuneable, thus opening a vast arena of applications where customizable reflectance is demanded. Of course, such an option is not unique for a layer of NPs; this is generally valid for any intermediate layer at the interface characterised by anisotropic permittivity tensor.

Similar angular dependence is also observed for NP array with larger lattice spacing of *a* = 5*R*, as shown in Fig. 8(d). These findings are consistent with the NP array of larger radius, *R* = 25 nm, with lattice spacing of *a* = 3*R* (see Fig. 8(b)) and of *a* = 5*R* (see Fig. 8(e)). For such lattice constants, we have shown the angular dependence of the reflectance maximum for both s- and p-polarised light for NPs of radii *R* = 6.25 nm (Fig. 8(c)) and *R* = 25 nm (Fig. 8(f)). We see that for s-polarised light the reflection maximum increases monotonically with increasing the angle of incidence *θ*. At any given angle, higher reflectance is found at shorter inter-particle spacing (*i.e.*, for *a* = 3*R*), which attributes to stronger plasmonic coupling^27^,^28^,^51^ between NPs. Reflectance is comparatively larger for the larger NPs even for a fixed ratio of lattice constant to NP radius, the reason behind which has been discussed in the previous sections.

The variation in the maximum reflectance level for p-polarised light is rather remarkable and not monotonous. With increase of the incident angle *θ*, the reflectance initially keeps on reducing, and goes to a minimum at a certain angle *θ* beyond which the reflectance again increases dramatically. That particular angle can be identified as Brewster’s angle at the interface of the NP embedding layer (layer 3) and the substrate (layer 4)^52^. When *θ* approaches the Brewster’s angle, reflectance gradually diminishes to zero at the Brewster’s angle where p-polarised light is forbidden. Beyond that angle, reflection of p-polarised light is permitted again, and the maximum reflectance level increases drastically with increasing *θ*, but with a spectral profile (not shown here) different to the typical bell shape. The effect of substrate permittivity on optical reflectance maximum and its corresponding wavelength at different light polarisation is further investigated as functions of incident angle (see Supplementary Figure S6).

These results, although practically relevant, are not surprising. Indeed, the effective medium theory analysed here maps the properties of a nanoscale system on an extra ‘Fresnel’ layer with however anisotropic polarisability and specific frequency dispersion. The system trends, therefore, should obey the general laws of geometric optics for layered systems^53^.

Thus, we show that the optical response of such plasmonic nanoparticle system can be tailored by tuning the NP size, lattice spacing, gap between NPs and the substrate, and optical constants of the materials constituting the ‘layers’. One can also achieve dynamic control of the system’s optical response by simply adjusting the polarisation and incident angle of impinging light. Recently other techniques have been proposed, based on coherent control of optical resonances in systems of plasmonic nanoparticles^54^,^55^. Such precise engineering of wavelength-selective optical reflectance/transmittance, powered by resonating plasmonic nanoparticles, could be promising for design and operation of future nano-optical devices.

## Discussion

A theoretical model—based on quasi-static dipolar approximations, image-charge theory, and the theory of multi-layer Fresnel reflection—studied in this work appears to fairly accurately describe the optical responses of two-dimensional arrays of plasmonic nanoparticles (NPs) at flat interface between two optically isotropic dielectric media (here the accuracy of the predictions of the model are tested by full-wave numerical simulations).

We provided explicit expressions to calculate optical responses for both square and hexagonal arrays of plasmonic nanoparticles. Considering the case of hexagonal lattice arrangement of gold nanospheres, which are immersed in water and assembled in the vicinity of a dielectric substrate, we investigated their reflectance spectra over a spectral range of 400 nm to 900 nm as functions of NP size, lattice constant, substrate permittivity, and distance of the NP layer from the interface (*i.e.*, from the top surface of the substrate).

We tested the generality of our model, and the results are depicted in Supplementary Figures S1–S5, which compare the theoretical reflectance spectra with those from simulations in all of the possible cases: Supplementary Figure S1 shows the results for small NPs, of 6.25 nm radius, in a high density array positioned either close or far from the substrate (gap = 1 nm and 5 nm); Supplementary Figure S2 displays the case of large NPs (with radius of 25 nm) in the scenario similar to those for small NPs in Fig. 3; Supplementary Figure S3 corresponds to large NPs (of 25 nm radius) in a high density array, placed close or far from the substrate (gap = 1 nm and 25 nm); Supplementary Figure S4 presents the case of large NPs (25 nm radius) in a low density array, positioned either close or far from the substrate (gap = 1 nm and 25 nm); Supplementary Figure S5 depicts the case of very large NPs with radius of 40 nm in both high and low density arrays while being positioned very close, moderately far, and far from the substrate (gap = 1 nm, 10 nm, and 50 nm). Using these figures we have quantified the range of validity of our model as functions of different system parameters.

Generally, the model is not very accurate for very-dense arrays of NPs with inter-NP spacing below 1–2 nm and for very large nanoparticles (with radii ≥ 40 nm) close to the interface. For NP arrays with inter-NP spacing of 3–4 nm or beyond, our theoretical model almost perfectly reproduces the simulation spectra. This makes our model very effective for practical usage, where NP capping ligands of typical lengths of 1.5–2 nm usually restrict inter-NP spacing to go below 3–4 nm. Even for very large NPs and for very-low density array, our model could provide a fairly reasonable qualitative match with the simulation.

The spectral trend of the peak wavelength 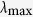, corresponding to the reflectance maximum, was found exhibiting blue-shift for a dielectric substrate material that could make the interface more reflective. With more reflection from the bare interface, it was interesting to observe contrasting trends in the cases of small and large NPs. With increase in substrate permittivity, the interface itself gets more reflective and for small NPs the overall reflectance was found to increase; whereas for large NPs reflectance decreased. These trends persist when the lattice constant of NP array is such that the NPs are strongly coupled to each other, for both types of systems with small and large gaps between the NP layer and the substrate. However, at large lattice constants the trend of maximum reflectance reverses for large NPs and even 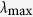 was found to follow an opposite trend of red-shift at large gap from the interface (Fig. 4).

It was observed that ‘enhanced transmission’ effect due to far-field constructive interference increases the transmission through the system at long wavelengths, even surpassing that from the bare interface (Fig. 5). Thus, it is found necessary to measure all three optical phenomena of reflection, transmission, and absorption to clearly appreciate the effects of parameters on the system’s overall optical responses. The effect of evanescent waves on NPs interacting with the substrate at short gap plays significant role in enhancing both reflection and transmission within visible spectrum; however, at long wavelengths in near infrared regime due to the enhanced transmission effect the trend in transmission profile reverses (Fig. 6).

At larger gaps (between NPs and the substrate), absorption in NPs increase drastically due to additional absorption of light (reflected from the interface), causing reflectance and transmittance to simultaneously reduce within visible spectra. At short gap, for a more reflective material as substrate, reflectance slightly increases until the absorption reaches its peak and beyond that peak wavelength reflectance reduces drastically. Transmittance, however, increases over the entire spectral range and the enhancement in transmission grows further at long wavelengths owing to the far-field constructive interference effect. At large gaps, where the evanescent waves of the NPs are weakly influenced by the presence of the substrate material, the trends of reflectance, transmittance and absorbance become opposite to those seen at short gaps. However, the enhancement in transmission is found to be larger than in the previous case, in the absence of strong confinement of light in the gaps (Figs 5 and 6).

Further, the study over wider range of system parameters on the reflectance maxima and peak wavelength 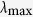 provided new means of tailoring the optical reflectance *at will*, by choosing appropriate lattice spacing, substrate material, and NP radius. Additionally, the reflectance from the NP system is found to be dynamically controllable by tailoring light incident angle and polarisation. Interestingly, it is also possible to minimize reflectance at will from the same physical setup by simply operating at large angle but with p-polarised light. These properties are thoroughly investigated considering different NP size and lattice constants to evaluate all possible practical scenarios (Figs 7 and 8). Such traits of dynamically altering the optical response of a given NP system would enable the application of this theory in building and optimizing smart optical metamaterials such as tuneable mirrors, windows, *etc.* in form of modern light reflecting and light harvesting devices.

## Additional Information

**How to cite this article**: Sikdar, D. and Kornyshev, A. A. Theory of tailorable optical response of two-dimensional arrays of plasmonic nanoparticles at dielectric interfaces. *Sci. Rep.* **6**, 33712; doi: 10.1038/srep33712 (2016).

## Supplementary Material

Supplementary Information

## Figures and Tables

**Table 1:** Parameters of the Drude-Lorentz model for gold.

*ε*_∞_	*ω*_p,D_ (eV)	*γ*_D_ (eV)	*s*_1_	*ω*_p1,L_ (eV)	*γ*_1,L_ (eV)	*s*_2_	*ω*_p2,L_ (eV)	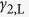 (eV)
5.9752	8.8667	0.03799	1.76	3.6	1.3	0.952	2.8	0.737
