# Supplementary material for: Theory of tailorable optical response of two-dimensional arrays of plasmonic nanoparticles at dielectric interfaces
Source: Sci Rep. 2016 Sep 22;6:33712. doi: 10.1038/srep33712 (PMC5031966; doi:10.1038/srep33712)
Supplement: Supplementary Information [file srep33712-s1.pdf]

SUPPORTING INFORMATION

Theory of tailorable optical response of two-dimensional arrays of plasmonic nanoparticles at dielectric interfaces

Debabrata Sikdar* and Alexei A. Kornyshev

Department of Chemistry, Faculty of Natural Sciences, Imperial College London,
Exhibition Road, South Kensington, London, SW7 2AZ, United Kingdom

Corresponding e-mails: *d.sikdar@imperial.ac.uk, a.kornyshev@imperial.ac.uk

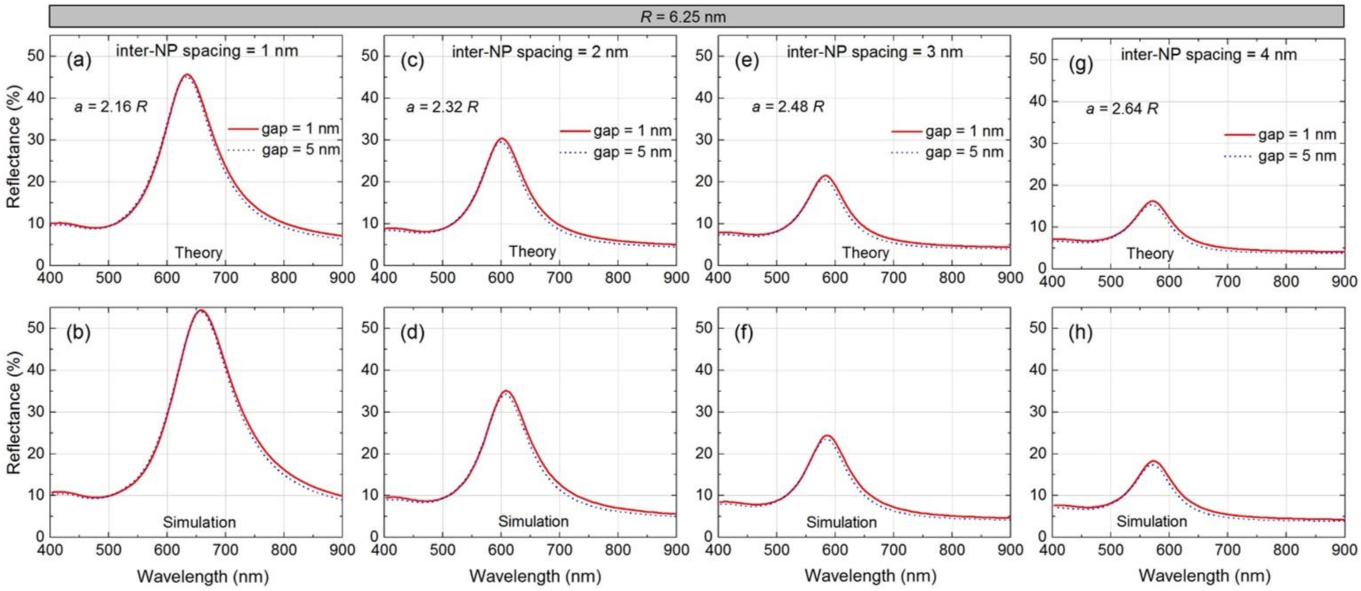

Figure S1. Comparison between two sets of optical reflectance spectra: one calculated using our theoretical model and the other based on numerical simulations—for small nanoparticles (NPs) with radius $R = 6.25$ nm. Considering hexagonal array of such gold nanospheres, the closeness of the theoretical model in predicting the simulated spectrum is depicted—at different lattice spacing and at different gaps of the NP layer from the substrate. Reflectance spectra are calculated for different lattice constant a (implying different inter-NP spacing) and for different distance (or gap) of the NP monolayer (layer 2) from the substrate (layer 4). In all cases we consider normal incidence of light *i.e.,* $\theta = 0^{\circ}$, $\varepsilon_1 = \varepsilon_3 = 1.78$ and $\varepsilon_4 = 4$.

Notice that even for such small NPs, intense plasmonic coupling among the NPs (at inter-NP spacing as small as 1 nm) leads to hybridized multipolar modes, which our dipolar quasi-static model fails to predict accurately [see (a) and (b)]. However, with increasing inter-NP spacing, the plasmonic coupling among the NPs gets weaker and the multipolar modes tend to cease to exist. In which case our dipolar model gets much closer towards matching the simulation results. Noticeably, at inter-NP spacing of 3 nm [see (e) and (f)] and beyond, the peak position and overall reflectance across the spectral range are found to be accurately calculated by the theoretical model. Considering such inter-NP spacing is of more practical significance as, in practice, each NP is usually capped with ligands (each having typical length of around 2 nm). This implies inter-NP spacing to be a minimum of around 4 nm, even for a high-density NP array. In this regime (or even for any larger inter-NP spacing) our theoretical model is found to be very accurate in estimating the optical response of such NP systems.

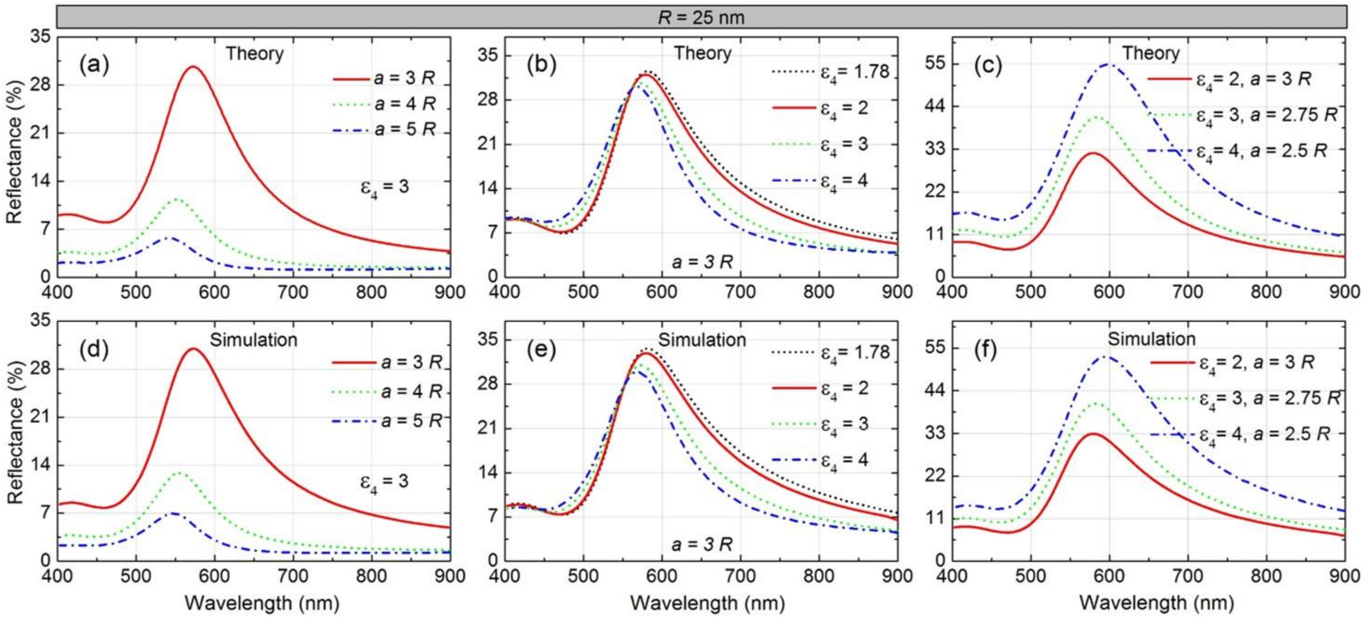

Figure S2. Effects of lattice spacing and substrate permittivity on optical reflection for NPs (Similar to Figure 3, but for relatively larger NPs with radius $R = 25$ nm). Reflectance spectrum calculated based on theory [(a)–(c)] and obtained from numerical simulations [(d)–(f)] as function of lattice constant [(a) and (d)] for a fixed substrate (layer 4) permittivity $\varepsilon_4 = 3$, as function of layer 4 permittivity ε_4 [(b) and (e)] for a fixed lattice constant $a = 3R$, and as function of both ε_4 and lattice constant a [(c) and (f)]. In all cases, the gold NPs are considered to be arranged in a 2D hexagonal lattice, where the gap between the NP layer and layer 4 substrate is fixed at 1 nm and $\varepsilon_1 = \varepsilon_3 = 1.78$.

Notice that there is an excellent agreement between theory and simulation results for relatively larger NPs at the lattice constants (or the inter-NP spacing) under consideration. Among all the cases considered here, the strongest inter-NP coupling takes place at lattice constant of $a = 2.5R$, where inter-NP spacing is of 12.5 nm. Here the plasmonic coupling mode is predominantly dipolar as can be correctly estimated by our dipolar quasi-static model. For similar or larger inter-NP spacing we could see that our model is pretty accurate. We further investigate the validity range of our model for much shorter inter-NP spacing, i.e., for very-dense NP arrays [see Figure S3].

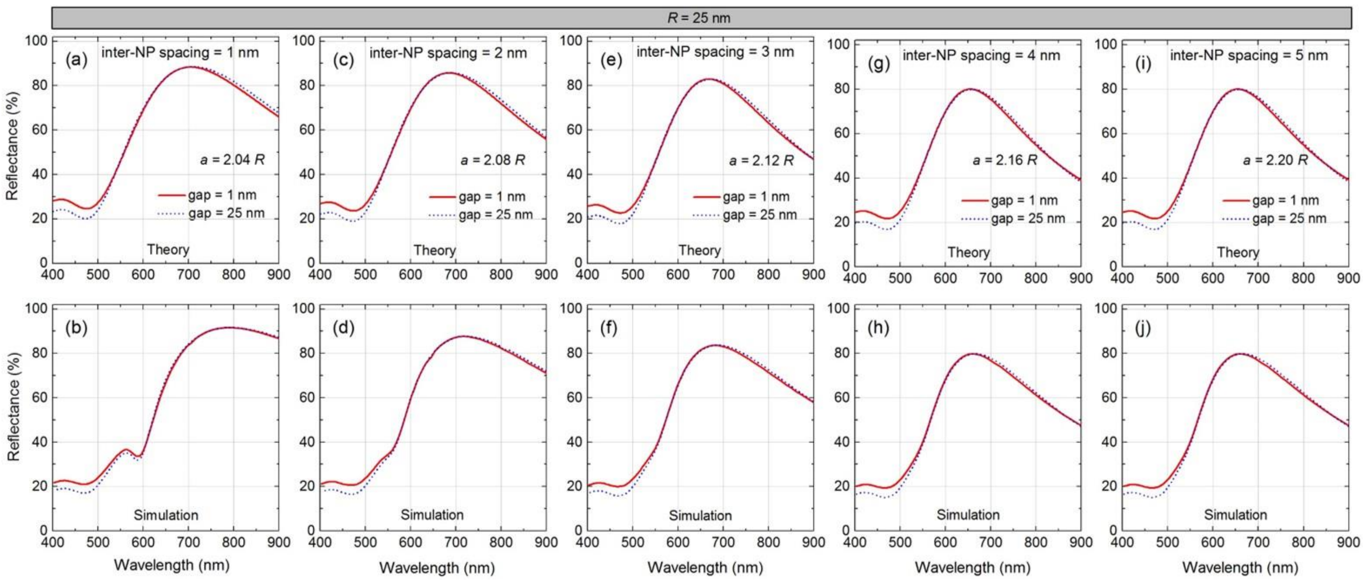

Figure S3. Comparison between two sets of optical reflectance spectra: one calculated using our theory and the other based on numerical simulations—for relatively large NPs of radius $R = 25$ nm in very-dense arrays. For very-dense hexagonal array of such gold nanospheres the closeness of the theoretical model in predicting the simulated spectrum is investigated at different lattice spacing and at different gaps of the nanoparticle (NP) layer from the substrate. Reflectance spectra are calculated for different lattice constant a (which provides the inter-NP spacing) and for different distance (or gap) of the NP monolayer (layer 2) from the substrate (layer 4). In all cases we consider normal incidence of light *i.e.,* $\theta = 0^{\circ}$, $\varepsilon_1 = \varepsilon_3 = 1.78$ and $\varepsilon_4 = 4$.

Notice that at inter-NP spacing as small as 1 nm (where inter-NP plasmonic coupling is very intense giving rise to hybridized multipolar modes) our dipolar quasi-static theory model fails to correctly predict dominant dipolar peak and the emergence of a high energy peak at around 550 nm, as seen in the simulation [see (a) and (b)]. With increasing inter-NP spacing [from 1 nm (a, b) to 5 nm (i, j)], plasmonic coupling among the NPs gets weaker, making multipolar modes and their contributions to disappear. This can be seen in form of the diminishing trend of the high energy peak and spectral overlap of the dominant peak position. This makes each theoretical spectrum to get closer to its corresponding one from simulation. As observed, from inter-NP spacing of 4 nm (g, h) the theoretical and simulated spectra comply very well to each other. This is of much practical significance, as the length of the ligands (capping the NPs) are typically around 2 nm. This ensures that even in a very dense array, two NPs could not usually get closer than 4 nm. The larger the lattice constant, the better is the match between theory and simulation. Although, a few minor discrepancies do exist even at such inter-NP spacing. These are mainly due to the fact that our theory does not include contributions from higher order modes, which could exist on individual NPs. Though not very significant, these high energy modes contribute to the optical responses to some extent, especially, through absorbance. Such minor differences lie mostly around the shorter wavelengths where the high energy modes of the NPs exist. Similar comparison between theory and simulation is also carried out for low density NP arrays [see Figure S4].

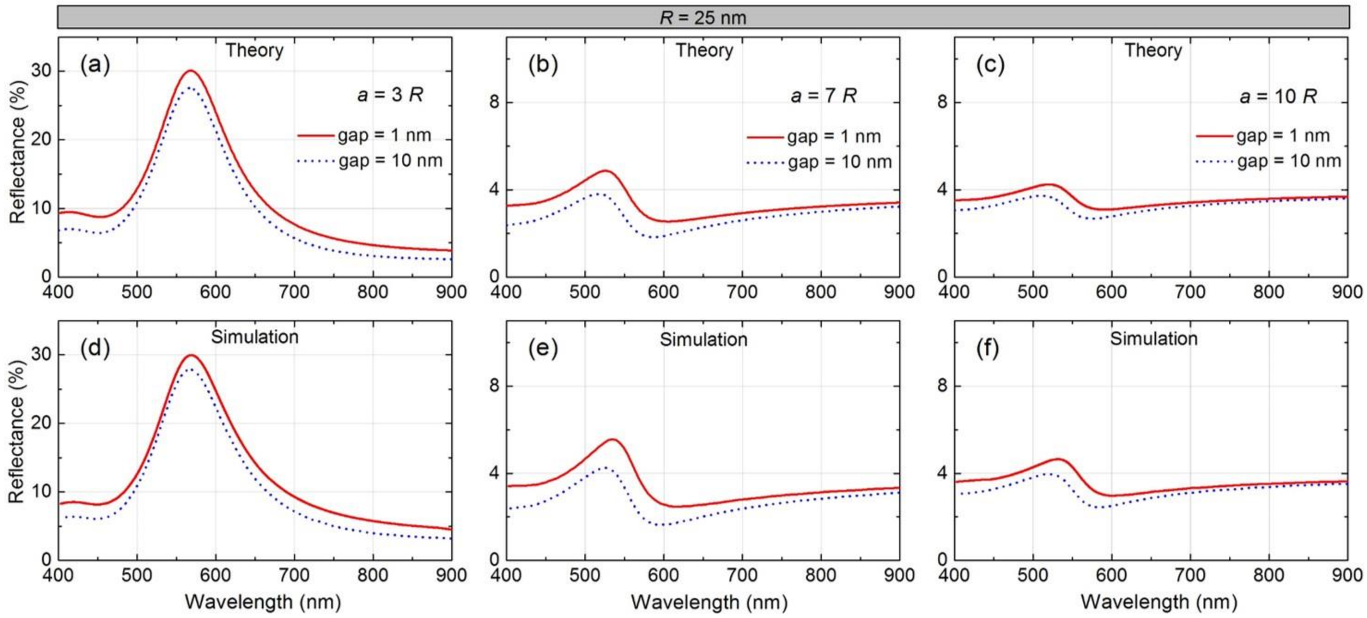

Figure S4. Comparison between theory and simulation in obtaining optical responses for low density arrays of nanoparticles (NPs)—for large NPs with radius $R = 25$ nm in low density arrays. In all cases we consider normal incidence of light *i.e.*, $\theta = 0^{\circ}$, $\varepsilon_1 = \varepsilon_3 = 1.78$ and $\varepsilon_4 = 4$. Optical reflectance spectra are calculated at different lattice spacing a and different gaps of the NP layer from substrate. Notice that there is an excellent match between our theoretical calculation and simulation results for low-density NP arrays, starting from $a = 3R$.

Here the inter-NP spacing is equal to the radius of each NP, implying inter-particle plasmonic coupling to be moderate. Such scenarios are accurately described by our quasi-static dipolar theory. However, at very low density arrays of NPs (as in the case of $a = 7R$ and $a = 10R$) there are a few minor discrepancies, which could be owing to the fact that our uniform thin metallic film approximation to replace the NP array may not be very accurate in such cases. Yet, the results give a decent prediction of the optical reflectance properties of those loosely packed NP arrays.

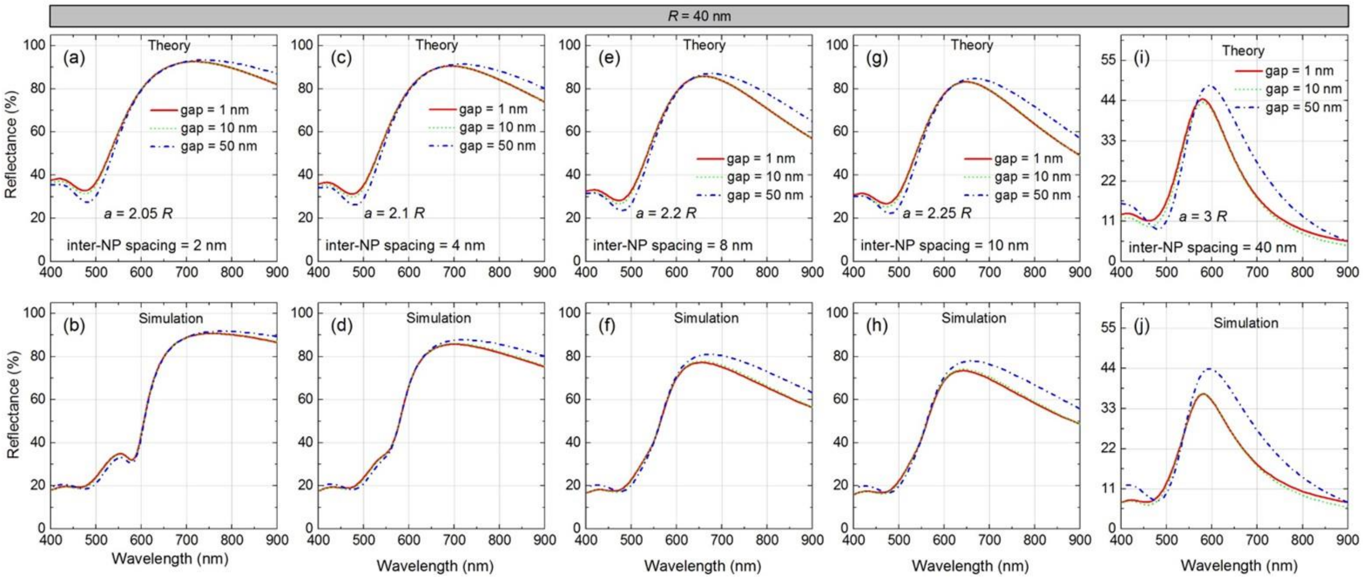

Figure S5. Comparison between two sets of optical reflectance spectra: one calculated using our theory and the other based on numerical simulations—for NPs with radius as large as $R = 40$ nm. For hexagonal array of such gold nanospheres the accuracy of the theoretical model in predicting the simulated spectrum is depicted at different lattice spacing and at different gaps of the nanoparticle (NP) layer from the substrate. Reflectance spectra are calculated for different lattice constant a (which provides the inter-NP spacing) and for different distance (or gap) of the NP monolayer (layer 2) from the substrate. In all cases we consider normal incidence of light *i.e.,* $\theta = 0^{\circ}$, $\varepsilon_1 = \varepsilon_3 = 1.78$ and $\varepsilon_4 = 4$.

Notice that at inter-NP spacing as small as 2 nm (where there is intense inter-NP plasmonic coupling giving rise to hybridized multipolar modes) our dipolar quasi-static theory model fails to predict the spectral characteristics, especially the high energy peak at around 550 nm, as seen in the simulation [see (a) and (b)]. With increasing inter-NP spacing, plasmonic coupling among the NPs gets weaker. This weakens the high energy peak, which then exhibits a diminishing trend, making the corresponding theoretical spectrum getting closer to those from simulation. The larger the lattice constant, the better is the match between theory and simulation. Here again, at inter-NP spacing of 4 nm and beyond, the long wavelength dipolar features get very accurately predicted by our quasi-static dipolar model. However, a minor discrepancies remain even at large lattice constants, which are due to the fact that each of these large individual NPs also possesses some higher order modes. The contributions of such modes are mainly through optical absorbance and not very significant. Hence, our theory can predict the optical responses of large NPs reasonably well in the regime discussed above.

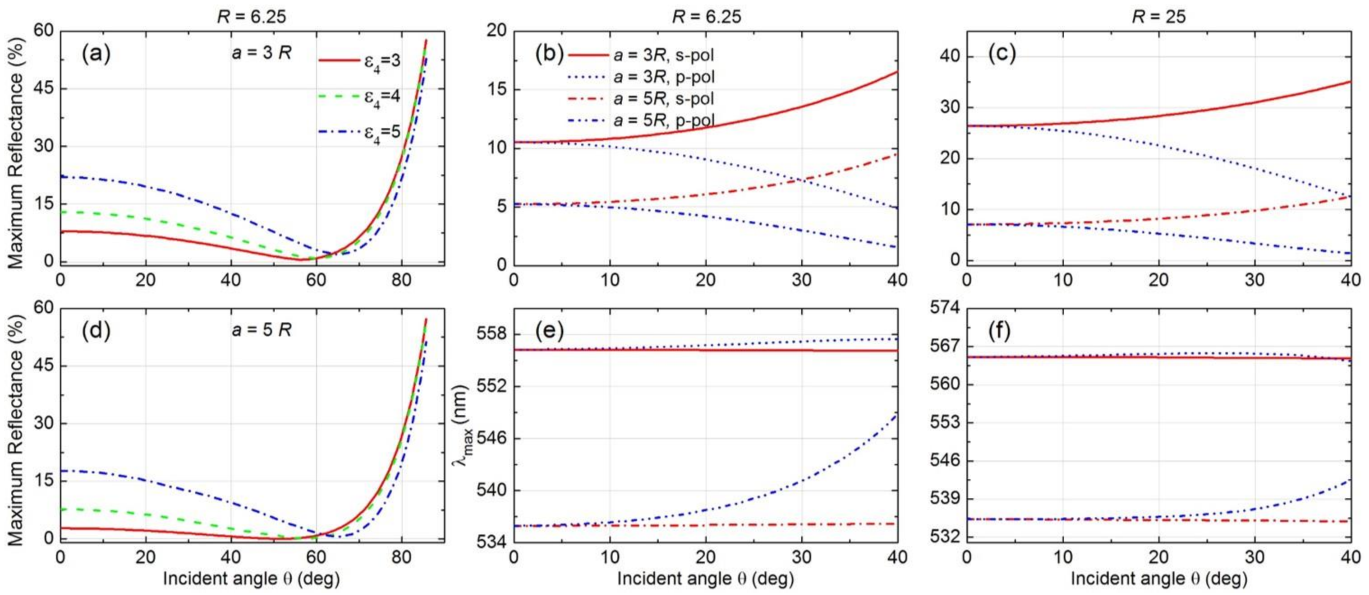

Figure S6. Variation in optical reflectance maximum and its corresponding wavelength at different substrate permittivity and light polarization as function of incident angle. Theoretically calculated maximum reflectance as function of incident angle θ for NP radius $R = 6.25$nm at different layer 4 permittivity ε_4 [(a) and (d)] for lattice constant $a = 3R$ (a) and $a = 5R$ (d). Maximum reflectance [(b) and (c)] and the corresponding wavelength $\lambda_{\max}$ [(e) and (f)] as functions of incident angle θ for NPs with radius $R = 6.25$nm [(b) and (e)] and $R = 25$nm [(c) and (f)] at different lattice constants and incident light polarization. In all cases, we consider $\varepsilon_1 = \varepsilon_3 = 1.78$, $\varepsilon_4 = 4$, and the gap between NP layer and substrate layer 4 as 1 nm.

The influence of substrate permittivity on Brewster's angle can also be inferred from figures 8(a) and 8(d), which plot the maximum reflectance from p-polarized light as a function of incident angle θ for different substrate permittivity ε_4. It is seen here that Brewster's angle increases with increase in ε_4. However, there is a negligible effect of lattice spacing on Brewster's angle. These findings are from theoretical calculations based on NP layer with NP radius of 6.25 nm. The same appears to be true for larger NP, of 25 nm radius, and hence they are not shown here. Figures 8(b)–8(f) present the variation in maximum reflectance and peak wavelength $\lambda_{\max}$ as functions of incident angle θ. Here, we restricted our study up to 40°, as at incident angle close to or beyond the Brewster's angle the spectral profile of reflectance gets distorted and is of less practical significance. It can be clearly seen that the maximum reflectance increases (decreases) for s- (p-) polarized light in both cases— for NP radii 6.25 nm [see figure 8(b)] and 25 nm [see figure 8(c)] at both short and large lattice constants. This clearly testifies the tuneable aspect of the reflectance spectrum as function of incident light characteristics for a system comprising plasmonic nanoparticles exhibiting plasmon-resonance enhanced optical properties.

Figures 8(e) and 8(f) show the changes in peak wavelength in each of the above cases, where for small NPs [see figure 8(e)] one can observe larger deviation between s- and p-polarized cases, increasing with θ, as compared

to large NPs [see figure 8(f)]. For p-polarization of incident light λ_{max} tends to red-shift, which signifies lowering of plasmonic coupling energy due to weakening of electric field component along the direction parallel to the NP layer with increase in incident angle. This effect is more prominent for smaller NPs and at large inter-particle spacing, as can be seen in figure 8(e) when compared to figure 8(f).
